# Hypoxia Combined With Interleukin‐17 Regulates Hypoxia‐Inducible Factor‐1α/Endothelial Nitric Oxide Synthase Expression in Pulmonary Artery Endothelial Cells

**DOI:** 10.1111/jcmm.70289

**Published:** 2025-01-17

**Authors:** Shuai Sun, Jianjun Mao, Yuan Ding, Lin Liu, Juanni Gong, Suqiao Yang, Jifeng Li, Tuguang Kuang, Ran Miao, Yuanhua Yang

**Affiliations:** ^1^ Department of Respiratory and Critical Care Medicine, Beijing Institute of Respiratory Medicine and Beijing Chao‐Yang Hospital Capital Medical University Beijing China; ^2^ Medical Research Center, Beijing Institute of Respiratory Medicine and Beijing Chao‐Yang Hospital Capital Medical University Beijing China

**Keywords:** chronic thromboembolic pulmonary hypertension, hypoxia, interleukin‐17, nitric oxide, pulmonary artery endothelial cell

## Abstract

The pathogenesis of chronic thromboembolic pulmonary hypertension may be multifactorial and requires further studies. We explored alterations in pulmonary artery endothelial cells under the hypoxic and elevated interleukin‐17 conditions that are commonly present in patients with chronic thromboembolic pulmonary hypertension. We measured the serum interleukin‐17 levels in 10 chronic thromboembolic pulmonary hypertension patients and 10 healthy control persons. The expressions and localisations of hypoxia‐inducible factor‐1α and endothelial nitric oxide synthase were detected in tissues. The levels of hypoxia‐inducible factor‐1α, endothelial nitric oxide synthase, nitric oxide, and reactive oxygen species in cultured pulmonary artery endothelial cells were examined under hypoxia and/or interleukin‐17 treatment. The serum interleukin‐17 level was increased in chronic thromboembolic pulmonary hypertension patients. Hypoxia‐inducible factor‐1α was increased, and endothelial nitric oxide synthase was decreased in chronic thromboembolic pulmonary hypertension pulmonary vascular tissue. After receiving the hypoxia combined with interleukin‐17 treatment, pulmonary artery endothelial cells showed increased levels of hypoxia‐inducible factor‐1α and phospho‐endothelial nitric oxide synthase (Thr495) (*p* = 0.001 and 0.063, respectively) and a decreased level of endothelial nitric oxide synthase (*p* < 0.001). In addition, the nitric oxide level was significantly decreased (*p* = 0.001), whereas the reactive oxygen species level was insignificantly increased in pulmonary artery endothelial cells. Chronic thromboembolic pulmonary hypertension patients might experience increased inflammation and hypoxia due to dysregulation of the hypoxia‐inducible factor‐1α/endothelial nitric synthase pathway in pulmonary artery endothelial cells under inflammation and hypoxia, contributing to the pathogenesis of chronic thromboembolic pulmonary hypertension.

## 1 Introduction

Chronic thromboembolic pulmonary hypertension (CTEPH) is characterised by pulmonary thromboembolism with a progressive increase in pulmonary artery pressure [1, 2]. Its pathogenesis is unknown but is considered to be multifactorial, such as the combination of pulmonary arterial thrombosis, intravascular hypoxia, and increased inflammation, with subsequent damage to the pulmonary artery endothelial cells (PAECs) [3, 4]. In the pulmonary endarterectomy (PEA) specimens of CTEPH patients, there were high expressions of various cytokines, such as interleukin‐6 (IL‐6), monocyte chemoattractant protein‐1, and interferon‐gamma‐induced protein‐10 (IP‐10), suggesting that inflammation could play a crucial role in the development of CTEPH [5]. Our preliminary result found that serum cytokine interleukin‐17 (IL‐17) expression was increased in patients with CTEPH. Considering that IL‐17 is an initiator of the early inflammatory response, which has been shown to play an essential role in the development of inflammatory bowel disease, asthma, and tuberculosis [6, 7], we believe that IL‐17 also participates in the pathogenesis and development of CTEPH, which warrants further studies.

PAECs are the innermost cells of pulmonary vessels, forming a protective barrier to the pulmonary vessels and having a role in the regulation of pulmonary vascular homeostasis. PAECs can produce endothelial nitric oxide synthase (eNOS), which has been demonstrated to regulate vasomotor function [8]. When PAECs are stimulated by hypoxia and inflammation, threonine at position 495 of eNOS is phosphorylated to form p‐eNOS (Thr495), which inhibits eNOS activation and nitric oxide (NO) production, leading to functional changes in the pulmonary artery endothelium, disruptions of pulmonary vascular integrity, increased vascular permeability, and dysfunction to the vasomotor dysfunction [3]. Hypoxia‐inducible factor‐1α (HIF‐1α) is a major regulator of gene expression in cells under hypoxic stress. HIF‐1α is expressed in a variety of cells and is associated with eNOS production [9]. Since IL‐17 is involved in the inflammatory response and its expression is increased in CTEPH, IL‐17 might participate in the regulation of HIF‐1α, eNOS, and reactive oxygen species (ROS) during the pathogenesis of CTEPH. However, this theory has never been studied previously.

Therefore, we performed the present study with the aim to investigating the role of the HIF‐1α/eNOS pathway in PAECs under the influences of hypoxia and inflammation and to exploring their roles in the development of CTEPH.

## 2 Materials and Methods

### 2.1 Study Setting and Ethical Consideration

The study was performed at the Beijing Chao‐Yang Hospital, China, between September 2019 and September 2023. The study protocol was approved by the hospital ethics committee (NO.2019‐ke‐377). Each of the study participants signed the written informed consent.

### 2.2 Collection of Blood Specimens

Peripheral blood specimens were collected from CTEPH patients (CTEPH group, *n* = 10) and healthy persons (control group, *n* = 10). Peripheral serum was harvested from peripheral blood after centrifugation.

### 2.3 Collection of Pulmonary Artery Tissue Specimens

Immunohistochemical staining was performed on specimens of pulmonary artery tissue collected during pulmonary endarterectomy of CTEPH patients (*n* = 3) and from the pulmonary arteries of lung transplant donor patients (*n* = 3). The western blot experiment was performed on specimens of pulmonary artery tissue collected during pulmonary endarterectomy in CTEPH patients (*n* = 4) and from the pulmonary arteries of lung transplant donors (*n* = 4).

### 2.4 Cell Culture

Human PAECs (ScienCell Research Laboratories, Carlsbad, CA, USA) were cultured in endothelial cell medium (ECM) (ScienCell) with the addition of 5% foetal bovine serum at 37°C with 5% CO_2_. When the cell confluence reached 80%–90%, cells underwent five passages before being used in the subsequent experiments.

Cells (5 × 10^5^ cells/T25‐cell culture bottles) were treated with 50 ng/mL IL‐17 dissolved in phosphate‐buffered saline (PBS) at 37°C for 24 h, under normoxic or hypoxic conditions. The in vitro hypoxic condition was induced in a hypoxic chamber (1% O_2_ and 5% CO_2_) for 24 h at 37°C. The cells in the control group had the same volume of PBS and were maintained at 37°C, with 21% O_2_ and 5% CO_2_ for 24 h.

### 2.5 Experimental Protocols

All of the laboratory experiments described in the experimental protocols were conducted in triplicate, and the data were analysed from at least three independent tests.

#### 2.5.1 Enzyme‐Linked Immunosorbent Assay

The Enzyme‐Linked Immunosorbent Assay Kit (Cloud‐Clone Corp., CN) was used to detect the IL‐17 levels in the peripheral blood specimens. Microplate wells were coated by recombinant IL‐17 protein. Lysates from serum (100 μL) or recombinant IL‐17 protein (100 μL), at the concentrations of 0, 15.6, 31.2, 65.2, 125, 250, 500, or 1000 pg/mL, were mixed and incubated with 100 μL anti‐IL‐17 monoclonal antibody for 60 min at 37°C. The mixed solution was loaded into the plate wells. After incubation at 37°C for 20 min, the stop reaction solution was added, and the plate was examined under a microplate reader to record the optical densities that were processed in CurveExpert 1.3 (Microsoft, Redmond, WA, USA).

#### 2.5.2 Immunohistochemistry Analysis

Immunohistochemical staining was accomplished following the standard protocol. The pulmonary artery tissues were deparaffinized in xylene, hydrated, and sliced into 5–7‐μm sections that were stained with antibodies against HIF‐1α (rabbit; 1:200 dilution), eNOS (mouse; 1:200 dilution), von Willebrand factor (vWF) (rabbit; 1:200 dilution), or α‐smooth muscle actin (α‐SMA) (rabbit; 1:200 dilution) antibodies using standard protocols. The anti‐αSMA and anti‐vWF antibodies were applied to identify smooth muscle cells and PAECs, respectively. Then, tissue sections were incubated with the secondary goat anti‐mouse antibodies, followed by imaging captured in light m microscopy at ×400 magnification. The colour depth represented the expression amount. We used continuous paraffin sections in this study. When HIF‐1α and vWF developed colour at the same location, HIF‐1α was considered to be expressed in the PAECs. If HIF‐1α and α‐SMA developed colour at the same location, HIF‐1α was considered to be expressed in the PAECs (eNOS was also observed according to this principle).

#### 2.5.3 Western Blot Analysis

The western blot analysis was performed following the standard protocol. Briefly, after treatment under normoxic or hypoxic conditions with or without IL‐17A at 37°C for 24 h, the cells were washed with PBS and lysed in the radioimmunoprecipitation assay buffer with protease inhibitor cocktail and 1 mM phenylmethylsulfonyl fluoride (both from Beyotime Biotechnology Inc., China) on ice for 30 min. The supernatant was collected after centrifugation at 13,400 *g* under 4°C for 15 min. Then, 15 μg of total protein was loaded onto the 10% sodium dodecyl sulphate polyacrylamide gel (Epizyme Biotechnology, China). After electrophoresis and protein transfer onto a polyvinylidene fluoride membrane (EMD Millipore, USA), the membrane was blocked and then probed by primary antibodies against HIF‐1α (rabbit; 1:1000 dilution), eNOS (mouse; 1:1000 dilution), p‐eNOS (Thr495) (rabbit; 1:1000 dilution), or β‐actin (mouse; 1:3000 dilution) at 4°C overnight. Finally, after rinsing with tris‐buffered saline with 0.1% Tween‐20 solution and staining with the horseradish peroxidase‐conjugated secondary antibody (1:3000 dilution) at room temperature for 1 h, the protein bands were inspected using the Western Blotting Luminol Reagent (Thermo Fisher Scientific Inc., USA).

#### 2.5.4 Quantitative Real‐Time Polymerase Chain Reaction

The reverse transcription was performed on total RNA extracted from PAECs (TRIzol reagent, Takara, Japan). The resultant cDNA was subjected to the quantitative real‐time polymerase chain reaction. The sequences of the primers used were HIF‐1α, forward 5′‐ACATTGAGAGCAAAGGGCT‐3′ and reverse 5′‐AGATGGTCAAGTTGGGAGC‐3′; and GAPDH, forward 5′‐GGAGCGAGATCCCTCCAAAAT‐3′ and reverse 5′‐GGCTGTTGTCATACTTCTCATGG‐3′.

#### 2.5.5 Nitric Oxide Assay

The supernatant from the PAECs culture was collected to measure the NO by the NO Griess reagent. A microplate reader (Thermo Fisher Scientific) was used to detect the NO at an optical density of 540 nm.

#### 2.5.6 Reactive Oxygen Species Assay

The pulmonary PAECs were treated under normoxic or hypoxic conditions with or without IL‐17A at 37°C for 24 h. A reactive oxygen species assay kit (cat. No. S0033; Beyotime Biotechnology) was used to determine the ROS released from PAECs. The intracellular ROS was detected by incubating the cells in serum‐free ECM with 10 mM 2,7‐dichlorofluorescein diacetate (DCFH‐DA) (Beyotime Biotechnology) or PBS (blank control) at 37°C in the dark for 30 min. The PAECs were then washed with cold PBS and incubated again in ECM with 1 mM DCFH‐DA at 37°C in the dark for an additional 30 min. Then, PAECs were trypsinised, washed, and resuspended in serum‐free ECM to a concentration of 1 × 10^6^ cells/L. The flow cytometry assay was performed with at least 10,000 events.

### 2.6 Statistical Analysis

Continuous data were tested for normality and expressed as the mean with standard deviation $\left(x\pm s\right)$ or the median with the interquartile range. Differences between groups were examined by the independent samples *t*‐test or rank sum test, depending on the normality test results. A *p* < 0.05 was considered statistically significant. All statistical analyses were performed using SPSS software (version 26.0; IBM, USA) or GraphPad Prism (version 8.0.2, GraphPad Software Inc., USA).

## 3 Results

### 3.1 Increase of Serum IL‐17 in Chronic Thromboembolic Pulmonary Hypertension Patients

**FIGURE 1:**
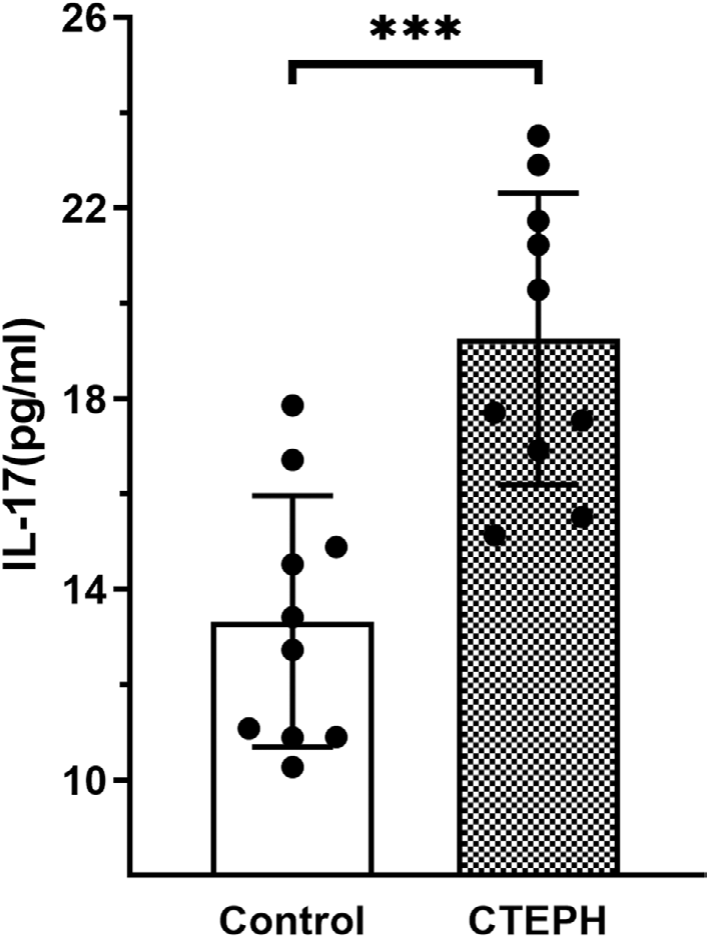
Enzyme‐linked immunosorbent assay of IL‐17 levels in serum samples from CTEPH patients and healthy control persons (CTEPH group, *n* = 10; control group, *n* = 10). Data are presented as the mean ± standard deviation. CTEPH, chronic thromboembolic pulmonary hypertension; ***p < 0.001.

The IL‐17 level in the serum of CTEPH patients (19.25 ± 3.06 pg/mL) was higher than that in the control group (13.33 ± 2.636 pg/mL), with a statistically significant difference (*p* < 0.001) (Figure 1).

### 3.2 Increase of HIF‐1α and Decrease of eNOS in the Pulmonary Vascular Tissue of Chronic Thromboembolic Pulmonary Hypertension Patients

**FIGURE 2:**
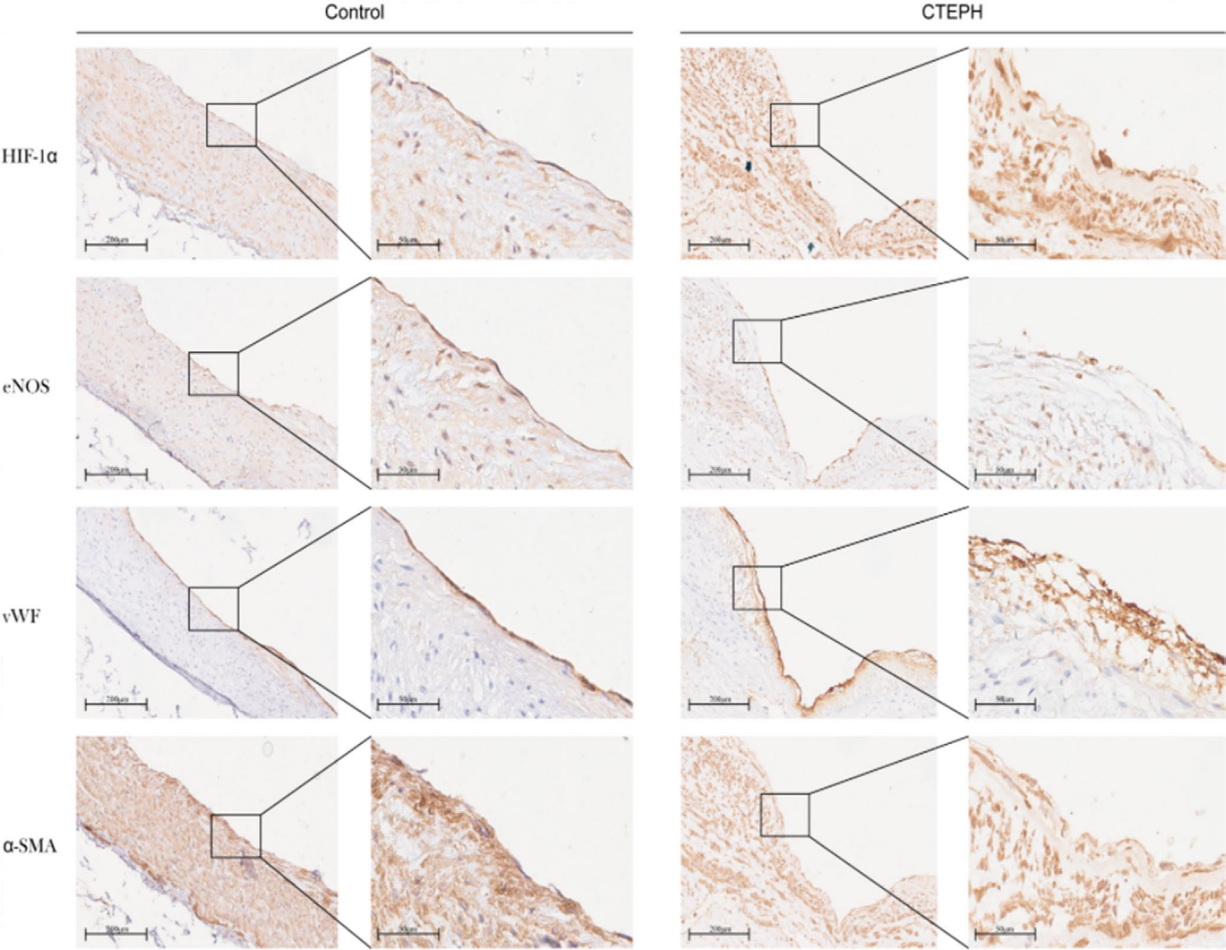
Immunohistochemistry staining of serially sectioned pulmonary artery tissues from CTEPH patients and lung transplant donors (control). The tissue sections were stained with HIF‐1α, eNOS, vWF, and α‐SMA antibodies, respectively (100× magnification; 400× magnification). Scale bars, 200, 50 μm. CTEPH, chronic thromboembolic pulmonary hypertension.

**FIGURE 3:**
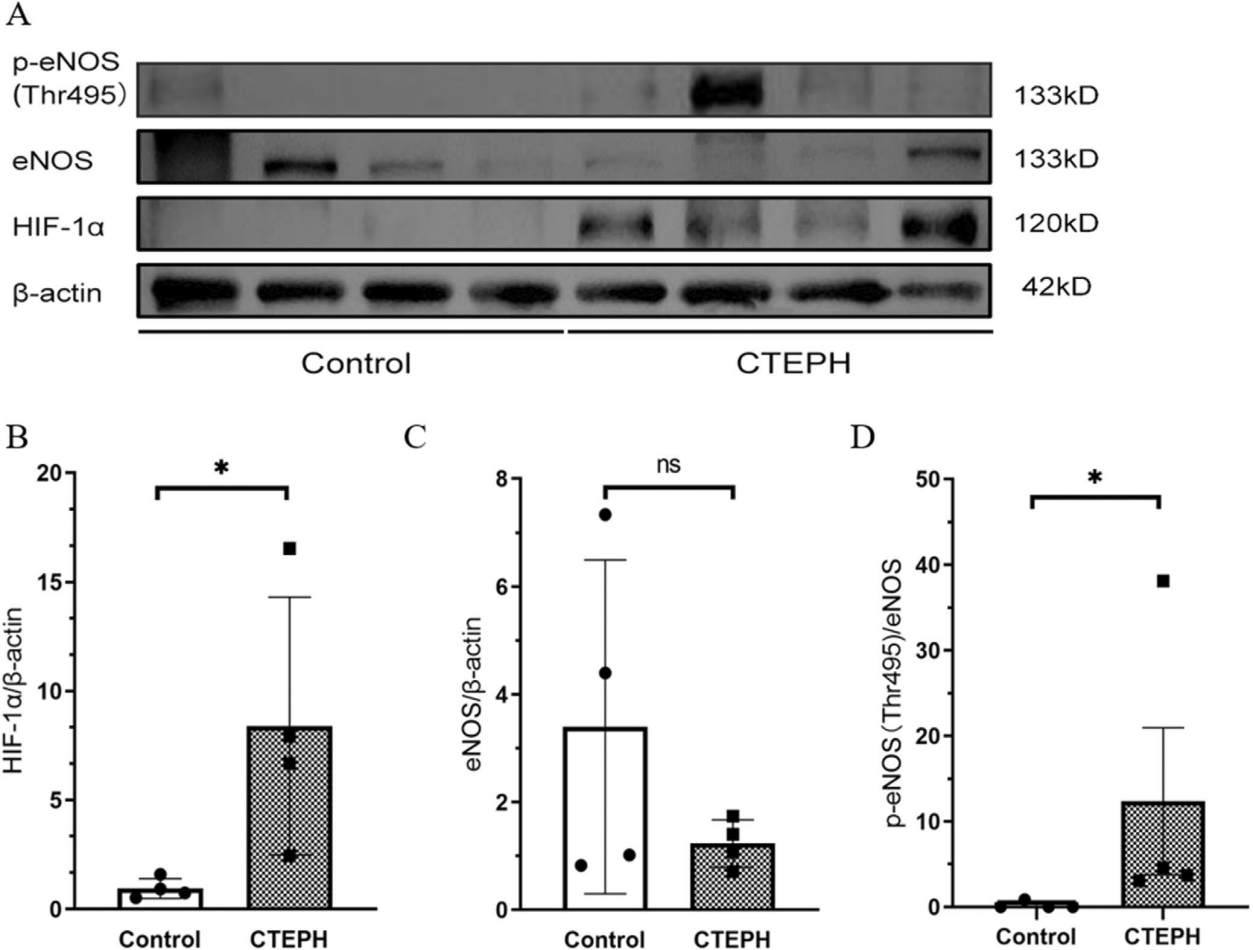
Western blot analysis of HIF‐1α and eNOS levels in pulmonary vascular tissues from CTEPH patients and controls. (A) HIF‐1α and eNOS levels in CTEPH patients and controls. (B–D) The ratio of greyscale values of target bands and β‐Actin bands. Data are expressed as the mean ± standard deviation. CTEPH, chronic thromboembolic pulmonary hypertension; *p < 0.05.

The baseline clinical characteristics of CTEPH patients are shown in Table 1. Immunohistochemistry staining of serially sectioned pulmonary artery tissues from CTEPH patients and lung transplant donors (control) is shown in Figure 2. The comparison between the pulmonary vascular tissues of lung transplant donors (control) and CTEPH patients showed that HIF‐1α expression increased in the pulmonary vascular tissues of CTEPH patients (*p* = 0.046), whereas eNOS expression decreased in the pulmonary vascular endothelium of CTEPH patients, although without a statistically significant difference (*p* = 0.216). In addition, eNOS‐Thr495 expression was significantly increased in CTEPH patients, with a statistically significant difference (*p* = 0.029) (Figure 3). These findings suggested that there were alterations in the expressions of HIF‐1α and eNOS in the pulmonary artery tissues of CTEPH patients. The change in p‐eNOS (Thr495) expression also indicated that there might have been a change in NO expression in CTEPH patients, which could have led to disrupted pulmonary vascular contractions.

**TABLE 1:** Basic Clinical Characteristics and Hemodynamics.

Variables	*n* = 4
Sex, male, *n* (%)	3 (75)
Age, year, M ± SD	62 ± 7.9
Smoking, *n* (%)	2 (50)
Family history of blood clots, *n* (%)	0 (0)
Venous thromboembolism, *n* (%)	3 (75)
Pulmonary embolism, *n* (%)	3 (75)
Body mass index, kg/m^2^, M ± SD	23.5 ± 1.6
WHO functional class (I‐II/ III‐IV), *n* (%)	3/1 (75/25)
Cardiac index, L/(min m^2^), M ± SD	2.4 ± 0.4
mean pulmonary artery pressure, mmHg, M ± SD	44.3 ± 10.2
6 min walking distance, meter, M ± SD	408.0 ± 101.8

Abbreviation: M ± SD, mean ± standard deviation.

### 3.3 Increase of HIF‐1α in Pulmonary Artery Endothelial Cells Under Hypoxia Combined With IL‐17 Intervention

**FIGURE 4:**
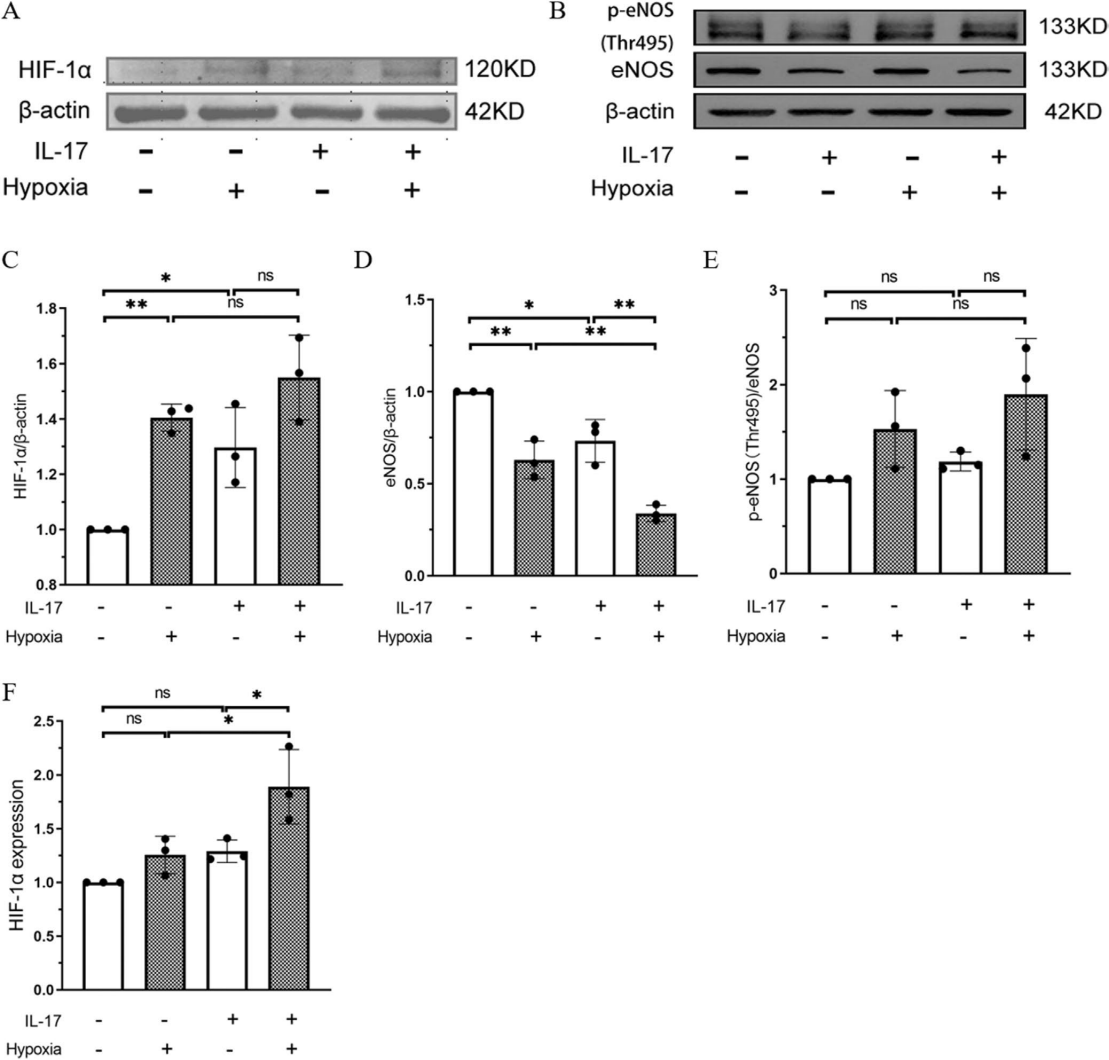
HIF‐1α and eNOS levels in pulmonary artery endothelial cells under different experimental conditions. (A, B) Western blot analysis of HIF‐1α and eNOS levels in PAECs. (C–E) The ratio of greyscale values of target bands and β‐Actin bands. (F) Real‐time polymerase chain reaction of the mRNA expression of HIF‐1α in PAECs. Data are expressed as the mean ± standard deviation. *p < 0.05, **p < 0.01; ns: No statistical differences.

The protein level of HIF‐1α of the hypoxic group was higher than that of the normoxic group, with a statistically significant difference (*p* = 0.008) (Figure 4A,C). The protein level of HIF‐1α was further increased after IL‐17 treatment under hypoxia when compared with that of the normal oxygen group, with a statistically significant difference (*p* = 0.001).

The mRNA level of HIF‐1α in the hypoxic group was higher than that of the normoxic group, although without a statistically significant difference (*p* = 0.450) (Figure 4F). After intervention with IL‐17 under hypoxic conditions, the mRNA level of HIF‐1α significantly increased (*p* = 0.003).

### 3.4 Decrease of eNOS Expression in Pulmonary Artery Endothelial Cells Under Hypoxia Combined With IL‐17 Intervention

The protein level of eNOS in the hypoxic group was lower than that of the normoxic group, with a statistically significant difference (*p* = 0.002) (Figure 4B,D). The p‐eNOS (Thr495)/eNOS expression level was higher in the hypoxic group than in the normoxic group, but without a statistically significant difference (*p* = 0.342) (Figure 4B,E). After combined IL‐17 treatment and hypoxia stimulation, the protein level of eNOS further decreased, with a statistically significant difference (*p* < 0.001), and p‐eNOS (Thr495)/eNOS expression further increased, but without a statistically significant difference (*p* = 0.063).

### 3.5 Decrease of Nitric Oxide in Pulmonary Artery Endothelial Cells Under Hypoxia Combined With IL‐17 Intervention

**FIGURE 5:**
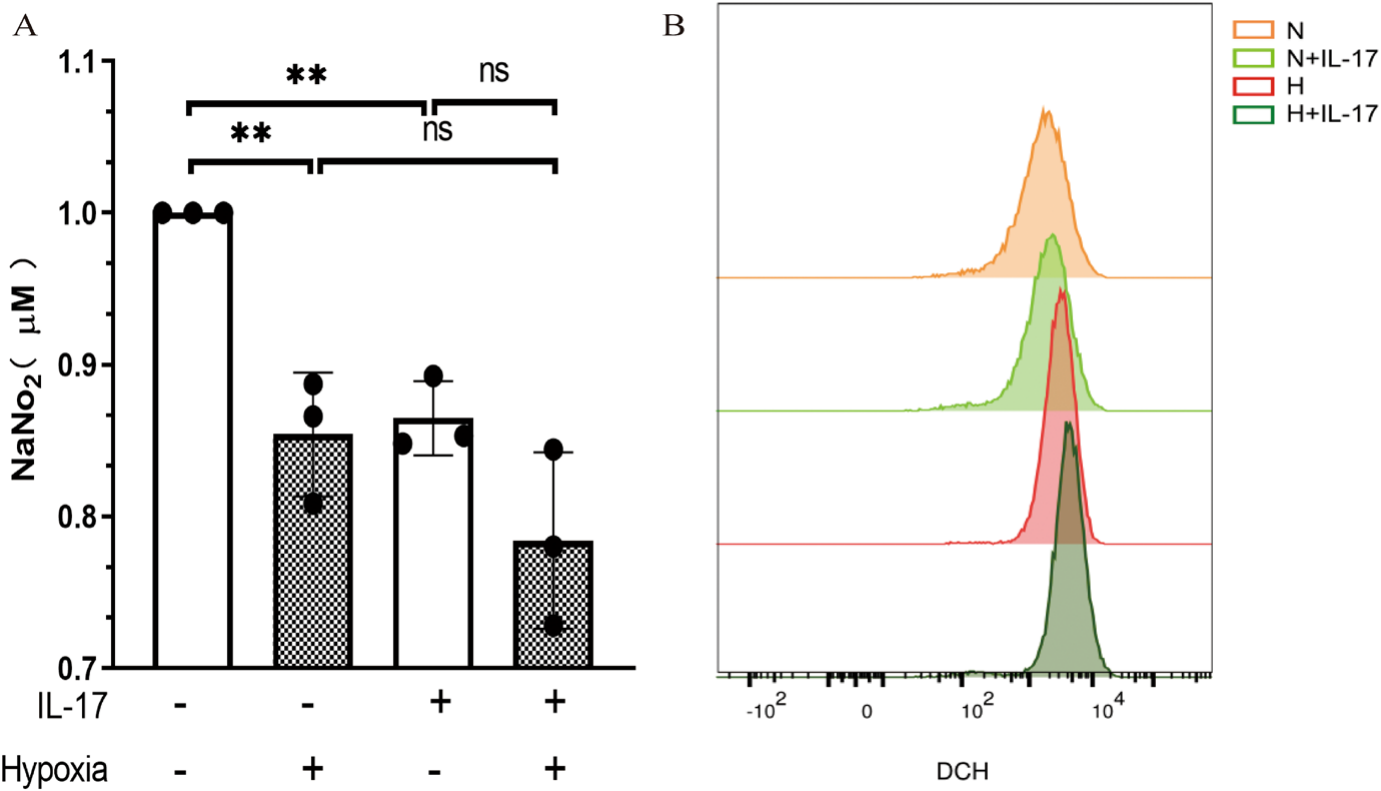
Nitric oxide assay of the amount of nitric oxide (A) and flow cytometry assay of the fluorescence intensity of reactive oxygen species (B) in pulmonary artery endothelial cells under different experimental conditions. Data are expressed as the mean ± standard deviation. **p < 0.01; ns: No statistical differences. DCH: 2′,7′‐dichlorofluorescein.

The amount of NO produced under hypoxia was lower than that under normoxia, with a statistically significant difference (*p* = 0.006) (Figure 5A). After the combined stimulation of hypoxia and IL‐17 treatment, the amount of NO was further reduced, with a statistically significant difference (*p* = 0.001).

### 3.6 Increase of Reactive Oxygen Species in Pulmonary Artery Endothelial Cells Under Hypoxia Combined With IL‐17 Intervention

The level of ROS in the hypoxic group was higher than that in the normoxic group. The level of ROS was further increased after IL‐17 treatment under the hypoxic condition (Figure 5B).

## 4 Discussion

CTEPH is a major type of pulmonary hypertension, characterised by undissolved or organised thrombus blocking pulmonary vessels with a progressive increase in pulmonary artery pressure [10]. In the current study, we showed that IL‐17 and HIF‐1α were increased and eNOS was decreased in CTEPH patients. After simultaneous treatment with hypoxia and IL‐17, there were increased levels of HIF‐1α and p‐eNOS and a decreased level of eNOS. Meanwhile, the NO decreased and ROS levels increased. All of these factors suggested that elevated levels of inflammation and hypoxia, along with dysregulated HIF‐1α/eNOS pathway, were involved in the pathogenesis of CTEPH. Our study results might imply a new treatment strategy in patients with CTEPH. To our knowledge, a similar study was never reported previously.

A registered study in Europe found that approximately 65.6% and 38.8% of patients with CTEPH had a history of acute pulmonary embolism or lower limb deep venous thrombosis, respectively [11, 12]. Acute pulmonary embolism can develop into chronic thromboembolic disease and CTEPH if there is incomplete thrombolysis or thrombus organisation [13]. A previous study reported that abnormal blood fibrinolysis, endothelial cell injury, angiogenesis disorders, hypoxic environments, inflammation, and immune factors could play a significant role in the pathogenesis of CTEPH, but the underlying molecular mechanisms were not fully understood [14, 15, 16].

IL‐17 is an important cytokine mainly produced by helper T cells. It usually functions as an initiator of early inflammatory response and can directly or indirectly induce the secretions of a variety of chemokines, cytokines, and inflammatory factors, thus mediating inflammation and immune response [17]. Inflammation and immune response play a significant role in thrombus remodelling and CTEPH development after acute pulmonary embolism [5, 18, 19, 20, 21]. Previous studies have reported that IL‐17 is involved in the pathogenesis of inflammatory bowel disease, psoriasis, and tumours [6, 7]. In the present study, we found the increased serum level of IL‐17 in patients with CTEPH. Considering the common role of IL‐17 as a trigger of the early inflammatory response and the importance of inflammatory response in the development of CTEPH, we believe that IL‐17 could participate in the pathogenesis of CTEPH.

Immunohistochemical staining of pulmonary artery intima tissue in CTEPH patients showed uneven and discontinuous endothelium [4], indicating the endothelial injury in these patients. The intima layer of the pulmonary artery is the innermost wall of pulmonary vessels and provides a protective barrier. An interrupted intima layer of pulmonary vessels will increase vascular permeability, with subsequent accumulation of inflammatory factors and disturbed vascular diastolic and contractile function [4]. A research centre in France successfully established a miniature pig model of CTEPH and verified that autotransfusion of endothelial progenitor cells could reduce the severity of pulmonary hypertension, probably through remodelling of cardiovascular structure [22]. A Japanese study also showed that riociguat, a guanylate cyclase stimulator for vasodilation, could induce endothelial progenitor cells to repair endothelial cells in patients with CTEPH [23]. All this evidence suggested the occurrence of endothelial cell injury in patients with CTEPH and that restoration of endothelial cell integrity could be a therapeutic approach for CTEPH.

Our study found that the expression of HIF‐1α was higher in pulmonary artery intima tissue of CTEPH patients than that of the control group. HIF‐1α is the main factor regulating gene expressions under hypoxia [24]. The increased expression of HIF‐1α in pulmonary intima indicated the hypoxic condition in the pulmonary vascular tissue of patients with CTEPH. HIF‐1α may be involved in the development of CTEPH.

Our study also showed that the level of eNOS was lower in the pulmonary artery intima tissue of CTEPH patients than that of the control group. In addition, the level of p‐eNOS (Thr495) was higher in CTEPH patients than in the control group. eNOS catalyses L‐arginine to produce NO and dilate pulmonary vessels. p‐eNOS (Thr495) loses its function and cannot stimulate NO production. When the activity of eNOS is inhibited and the content of NO decreases, the blood vessels contract and intravascular pressure increases, leading to CTEPH.

Since IL‐17 is involved in the initiation of inflammatory response and HIF‐1α is involved in the hypoxic reactions, the altered levels of IL‐17 and HIF‐1α in our study suggested that the inflammation and hypoxia might jointly regulate the development of CTEPH. Therefore, we explored the combined effects of IL‐17 and hypoxia in cultured PAECs in vitro. We found that increased HIF‐1α and p‐eNOS (Thr495) with decreased eNOS after the hypoxia stimulation combined with IL‐17 treatment suggested that hypoxia combined with IL‐17 stimulation might play a role in regulating the expressions of HIF‐1α and eNOS in PAECs. In order to explore whether eNOS could regulate the production of NO, we further measured the amount of NO in the supernatant of cell culture, confirming the decreased NO level under the hypoxic and inflammatory conditions.

We then explored the ROS level after the combined intervention of hypoxia and IL‐17. The results showed that the production of ROS increased after the intervention of hypoxia and IL‐17. In the mouse model of acute pulmonary hypertension, there was functional damage of PAECs with increased production of nicotinamide adenine dinucleotide phosphate (NADPH) oxidase. The increased NADPH oxidase could result in excessive ROS production and oxidative stress to PAECs, with subsequent changes in the function of PAECs [25]. In our study, the increased production of ROS indicated that oxidative stress occurred in PAECs, which might be one of the pathogenesis pathways of CTEPH. Previous studies have shown that the imbalance of NO production is one of the main causes of pulmonary hypertension. When ROS production increases, consumption of NO will make eNOS uncoupling at the same time [26, 27]. Our study showed decreased eNOS and NO, as well as increased p‐eNOS (Thr495), in human PAECs treated with hypoxia and IL‐17. These changes might be related to the increased ROS production, which necessitates further exploration of the occurrence mechanism of CTEPH.

## 5 Conclusion

Hypoxia combined with IL‐17 resulted in increased HIF‐1α expression, decreased eNOS expression, decreased NO, and increased ROS expression in PAECs, which might contribute to the pathogenesis of CTEPH.

## Author Contributions

**Shuai Sun:** data curation (lead), formal analysis (lead), methodology (lead), software (lead), visualization (lead), writing – original draft (lead). **Jianjun Mao:** writing – review and editing (equal). **Yuan Ding:** writing – review and editing (equal). **Lin Liu:** writing – review and editing (equal). **Juanni Gong:** writing – review and editing (equal). **Suqiao Yang:** writing – review and editing (equal). **Jifeng Li:** writing – review and editing (equal). **Tuguang Kuang:** writing – review and editing (equal). **Ran Miao:** project administration (lead), supervision (lead), writing – review and editing (lead). **Yuanhua Yang:** funding acquisition (supporting), project administration (supporting), writing – review and editing (supporting).

## Ethics Statement

The study was performed at Beijing Chao‐yang Hospital, Beijing, China. The study protocol was approved by the hospital ethics committee (NO.2019‐ke‐377).

## Conflicts of Interest

The authors declare no conflicts of interest.

## Data Availability

The authors have nothing to report.

## References

[1] L. Yan , X. Li , Z. Liu , et al., “Research Progress on the Pathogenesis of CTEPH,” Heart Failure Reviews 24, no. 6 (2019): 1031–1040, 10.1007/s10741-019-09802-4. 31087212

[2] M. Tomaszewski , E. Grywalska , W. Topyła‐Putowska , et al., “High CD200 Expression on T CD4+ and T CD8+ Lymphocytes as a Non‐Invasive Marker of Idiopathic Pulmonary Hypertension–Preliminary Study,” Journal of Clinical Medicine 10, no. 5 (2021): 50950, 10.3390/jcm10050950. PMC7957729 33804413

[3] Y. J. Jin , R. Chennupati , R. Li , et al., “Protein Kinase N2 Mediates Flow‐Induced Endothelial NOS Activation and Vascular Tone Regulation,” Journal of Clinical Investigation 131, no. 21 (2021): 5734, 10.1172/JCI145734. PMC8553558 34499618

[4] O. Tura‐Ceide , V. Smolders , N. Aventin , et al., “Derivation and Characterisation of Endothelial Cells From Patients With Chronic Thromboembolic Pulmonary Hypertension,” Scientific Reports 11, no. 1 (2021): 18797, 10.1038/s41598-021-98320-1. 34552142 PMC8458486

[5] D. Zabini , A. Heinemann , V. Foris , et al., “Comprehensive Analysis of Inflammatory Markers in Chronic Thromboembolic Pulmonary Hypertension Patients,” European Respiratory Journal 44, no. 4 (2014): 951–962, 10.1183/09031936.00145013. 25034560

[6] L. Wang , J. Liu , W. Wang , et al., “Targeting IL‐17 Attenuates Hypoxia‐Induced Pulmonary Hypertension Through Downregulation of β‐Catenin,” Thorax 74, no. 6 (2019): 564–578, 10.1136/thoraxjnl-2018-211846. 30777899

[7] M. J. McGeachy , D. J. Cua , and S. L. Gaffen , “The IL‐17 Family of Cytokines in Health and Disease,” Immunity 50, no. 4 (2019): 892–906, 10.1016/j.immuni.2019.03.021. 30995505 PMC6474359

[8] A. N. le , S. S. Park , M. X. le , et al., “DRG2 Depletion Promotes Endothelial Cell Senescence and Vascular Endothelial Dysfunction,” International Journal of Molecular Sciences 23, no. 5 (2022): 2877, 10.3390/ijms23052877. 35270019 PMC8911374

[9] J. Cheng , H. L. Yang , C. J. Gu , et al., “Melatonin Restricts the Viability and Angiogenesis of Vascular Endothelial Cells by Suppressing HIF‐1α/ROS/VEGF,” International Journal of Molecular Medicine 43, no. 2 (2019): 945–955, 10.3892/ijmm.2018.4021. 30569127 PMC6317691

[10] I. M. Lang , I. A. Campean , R. Sadushi‐Kolici , R. Badr‐Eslam , C. Gerges , and N. Skoro‐Sajer , “Chronic Thromboembolic Disease and Chronic Thromboembolic Pulmonary Hypertension,” Clinics in Chest Medicine 42, no. 1 (2021): 81–90, 10.1016/j.ccm.2020.11.014. 33541619

[11] E. Mahmud , M. M. Madani , N. H. Kim , et al., “Chronic Thromboembolic Pulmonary Hypertension: Evolving Therapeutic Approaches for Operable and Inoperable Disease,” Journal of the American College of Cardiology 71, no. 21 (2018): 2468–2486, 10.1016/j.jacc.2018.04.009. 29793636

[12] S. Guth , A. M. D'Armini , M. Delcroix , et al., “Current Strategies for Managing Chronic Thromboembolic Pulmonary Hypertension: Results of the Worldwide Prospective CTEPH Registry,” ERJ Open Research 7, no. 3 (2021): 850, 10.1183/23120541.00850-2020. PMC8365143 34409094

[13] N. H. Kim , M. Delcroix , X. Jais , et al., “Chronic Thromboembolic Pulmonary Hypertension,” European Respiratory Journal 53, no. 1 (2019): 1801915, 10.1183/13993003.01915-2018. 30545969 PMC6351341

[14] M. L. Bochenek , N. S. Rosinus , M. Lankeit , et al., “From Thrombosis to Fibrosis in Chronic Thromboembolic Pulmonary Hypertension,” Thrombosis and Haemostasis 117, no. 4 (2017): 769–783, 10.1160/TH16-10-0790. 28150849

[15] E. M. Neto‐Neves , M. B. Brown , M. V. Zaretskaia , et al., “Chronic Embolic Pulmonary Hypertension Caused by Pulmonary Embolism and Vascular Endothelial Growth Factor Inhibition,” American Journal of Pathology 187, no. 4 (2017): 700–712, 10.1016/j.ajpath.2016.12.004. 28183533 PMC5397717

[16] X. Liu , H. Zhou , and Z. Hu , “Resveratrol Attenuates Chronic Pulmonary Embolism‐Related Endothelial Cell Injury by Modulating Oxidative Stress, Inflammation, and Autophagy,” Clinics 77 (2022): 100083, 10.1016/j.clinsp.2022.100083. 35932505 PMC9357834

[17] S. P. D. Berry , C. Dossou , A. Kashif , et al., “The Role of IL‐17 and Anti‐IL‐17 Agents in the Immunopathogenesis and Management of Autoimmune and Inflammatory Diseases,” International Immunopharmacology 102 (2022): 108402, 10.1016/j.intimp.2021.108402. 34863654

[18] N. Skoro‐Sajer , C. Gerges , M. Gerges , et al., “Usefulness of Thrombosis and Inflammation Biomarkers in Chronic Thromboembolic Pulmonary Hypertension‐Sampling Plasma and Surgical Specimens,” Journal of Heart and Lung Transplantation 37, no. 9 (2018): 1067–1074, 10.1016/j.healun.2018.04.003. 29802084

[19] G. Simonneau , A. Torbicki , P. Dorfmüller , and N. Kim , “The Pathophysiology of Chronic Thromboembolic Pulmonary Hypertension,” European Respiratory Review 26, no. 143 (2017): 160112, 10.1183/16000617.0112-2016. 28356405 PMC9488693

[20] F. Gaertner and S. Massberg , “Blood Coagulation in Immunothrombosis‐At the Frontline of Intravascular Immunity,” Seminars in Immunology 28, no. 6 (2016): 561–569, 10.1016/j.smim.2016.10.010. 27866916

[21] D. van Uden , T. Koudstaal , J. A. C. van Hulst , et al., “Evidence for a Role of CCR6+ T Cells in Chronic Thromboembolic Pulmonary Hypertension,” Frontiers in Immunology 13 (2022): 861450, 10.3389/fimmu.2022.861450. 35572511 PMC9094486

[22] F. Loisel , B. Provost , J. Guihaire , et al., “Autologous Endothelial Progenitor Cell Therapy Improves Right Ventricular Function in a Model of Chronic Thromboembolic Pulmonary Hypertension,” Journal of Thoracic and Cardiovascular Surgery 157, no. 2 (2019): 655–666, 10.1016/j.jtcvs.2018.08.083. 30669226

[23] K. Yamamoto , R. Nishimura , F. Kato , et al., “Protective Role of Endothelial Progenitor Cells Stimulated by Riociguat in Chronic Thromboembolic Pulmonary Hypertension,” International Journal of Cardiology 299 (2020): 263–270, 10.1016/j.ijcard.2019.07.017. 31337550

[24] J. W. Lee , J. Ko , C. Ju , and H. K. Eltzschig , “Hypoxia Signaling in Human Diseases and Therapeutic Targets,” Experimental & Molecular Medicine 51, no. 6 (2019): 1–13, 10.1038/s12276-019-0235-1. PMC6586801 31221962

[25] M. Brandt , E. Giokoglu , V. Garlapati , et al., “Pulmonary Arterial Hypertension and Endothelial Dysfunction Is Linked to NADPH Oxidase‐Derived Superoxide Formation in Venous Thrombosis and Pulmonary Embolism in Mice,” Oxidative Medicine and Cellular Longevity 2018 (2018): 1860513, 10.1155/2018/1860513. 29983855 PMC6015670

[26] A. Jaitovich and D. Jourd'heuil , “A Brief Overview of Nitric Oxide and Reactive Oxygen Species Signaling in Hypoxia‐Induced Pulmonary Hypertension,” Advances in Experimental Medicine and Biology 967 (2017): 71–81, 10.1007/978-3-319-63245-2_6. 29047082 PMC5863727

[27] D. Xu , Y. H. Hu , X. Gou , et al., “Oxidative Stress and Antioxidative Therapy in Pulmonary Arterial Hypertension,” Molecules 27, no. 12 (2022): 3724, 10.3390/molecules27123724. 35744848 PMC9229274

